# Isolation of 60 strains from fermented milk of mares and donkeys in Algeria and identification by 16S rRNA sequencing of lactobacilli: Assessment of probiotic skills of important strains and aromatic productivity power

**DOI:** 10.14202/vetworld.2024.829-841

**Published:** 2024-04-15

**Authors:** Fouzia Benameur, Kawthar Belkaaloul, Omar Kheroua

**Affiliations:** Laboratory of Physiology of Nutrition and Food Safety, Department of Biology, Faculty of Natural and Life Sciences, University Oran 1 Ahmed Ben Bella, Oran, Algeria

**Keywords:** Algeria, aromatic productivity, lactic acid bacteria, mare and donkey milk, probiotic skills

## Abstract

**Background and Aim:**

Donkey and mare milk have high nutritional and functional values, but their lactic acid bacteria (LAB) content remains poorly studied and undervalued in the Algerian dairy industry. This study aimed to isolate and select LAB strains that produce antimicrobial substances during fermentation and to characterize the probiotic profiles of each extracted strain to indicate their potential for antioxidant and proteolytic activity.

**Materials and Methods:**

This study focuses on isolating and identifying lactic acid bacterial strains from 10 Equid-fermented milk samples collected in two regions of El Bayed Wilaya (Algeria). Identification of LAB strains was obtained by 16S rRNA sequencing. The probiotic properties of important strains and their aromatic productivity power are assessed. To evaluate their antibacterial activity against *Listeria monocytogenes*, *Staphylococcus aureus*, *Chryseobacterium joostei, Pseudomonas aeruginosa*, and *Escherichia coli*, we selected 21 strains. Different induction methods have been used to amplify the antibacterial effects against these pathogenic strains.

**Results:**

Among a total of 60 identified strains, 31 had a probiotic profile, and most were catalase-negative. Aromatic productivity power was observed in eight strains: *Lactiplantibacillus plantarum*, *Lactobacillus casei*, *Lactobacillus paracasei*, *Weissella* *confusa*, *Weissella cibaria*, *Leuconostoc mesenteroides*, *Leuconostoc lactis*, and *Lactobacillus* sp1.

**Conclusion:**

Our results provide insight into the considerable diversity of LAB present in fermented donkey and mare milk. To meet the expectations of the Algerian dairy industry, it is important that the probiotic skills of the nine selected strains are met. In addition, a significant number of these strains may have important probiotic activity and biotechnological potential.

## Introduction

In the past, curd was processed in an artisanal and traditional way to extend its shelf life. It has recently become a popular food in healthy daily diet. Fermented milk products currently play an important nutritional role in modern life. Fermented dairy products are known to be good vectors of probiotics, in particular, because they are widely consumed. To enrich and diversify the spectrum of lactic acid bacteria (LAB) from Equid milk in Algeria, it is necessary to identify new strains of LAB. The nutritional and therapeutic properties of horse milk have been known since ancient times, and horses are traditionally milked in the Central Asia and Eastern Europe, where koumiss and other fermented horse milk products with claimed health benefits are produced [1]. Compared to horses, donkeys evolved under different environmental conditions [2]. For a long time, donkey milk has been recognized as a common remedy. More recently, horse colostrum and milk production have been developed in Europe [3, 4] and then spread throughout Asia. In North Africa, equid milk is recognized as a therapeutic food as well as a highly appreciated cosmetic product [5]. In the last few decades, donkey milk has attracted increasing research interest. Although extensive reviews on the composition of equid milk [1, 5–8] are available, many problems remain related to the stability and functionality of probiotics in equid dairy products.

Equid’s milk for cosmetic products is more accessible than fresh bovine milk in Europe and Asia but not in North Africa. It has a remarkable similarity to human milk and, in addition to its high nutritional content, it contains a large number of immune factors. Since Egyptian antiquity, donkey’s milk has had food and cosmetic uses. It is rich in sugars and phosphates, low in caseins, and it is regarded as a rare and valuable product in Algeria and around the world. Following climate change, the world’s milk reserves have mainly reduced and become scarce in some North African countries, making pastures unfavorable for milk-producing mammals [4, 9–13].

LAB is the basis for fermented milk and the production of cheeses, yogurts, and many other types of cheese [14]. The main function of these bacteria is to reduce the pH of fermented milk at the expense of lactose in accordance with the kinetics specific to each production process. Proteolysis due to LAB makes it possible to obtain short peptides and free amino acids, which are precursors for many flavoring products [14]. A large number of LAB strains have probiotic functions that are beneficial to the host if they are taken in sufficient quantities. The viability of probiotic bacteria is important for the survival of foods during shelf life and transit in acidic stomach. To screen probiotic bacteria, they must also be resistant to degradation by hydrolytic enzymes and bile salts in the small intestine [15–17].

This study aimed to isolate and select LAB strains that produce antimicrobial substances during fermentation from donkey and mare’s milk. The characterization and probiotic profiles of each extracted strain and their potential for antioxidant and proteolytic activity are highlighted.

## Materials and Methods

### Ethical approval

This study was approved by the Algerian Institutional Ethics Committee for Animal Research (approval ID: 45/DGLPAG/DVA. SDA.14) and supported by the Ministry of Higher Education and Scientific Research-Algeria and by the PRFU Project (D00L01UN310120220001). Milk collection and animal restraint were carried out under the supervision of a veterinarian. All microbiological tests were carried out at the Laboratory of Physiology of Nutrition and Food Safety, Department of Biology, Faculty of Nature and Life Sciences, Oran 1 Ahmed Ben Bella University.

### Study period and location

The current study was conducted according to the ethical guidelines of the Laboratory of Nutrition Physiology and Food Safety, Department of Biology, Faculty of Nature and Life Sciences, University Oran 1 Ahmed Ben Bella, Oran, Algeria

### Sampling area and culture medium

Milk samples from mares and donkeys were obtained from different sites in El-Bayad Wilaya (Algeria). Five samples of mare’s milk and five samples of donkey’s milk were obtained from Lebyad Sid Cheikh, located 100 km South-east of El-Bayad, and Chegig, located 30 km South-west of El-Bayad. Milk samples were stored in sterile bottles at 4°C during transport and placed in an oven at 37°C for fermentation.

Selective isolation of LAB strains was performed on MRS (deMan, Ragosa, Sharpe) medium (pH 6.5) for lactobacilli and M17 medium (pH 6.5) for lactic hulls.

One ml of milk was taken from each sample, and the decimal dilution method was used for 10^-1^–10^-7^ in physiological water. One milliliter of each of the last three dilutions (10^-5^, 10^-6^, and 10^-7^) was deeply inoculated into the acidified MRS media. Petri dishes were incubated at 30°C for 24 h.

Microscopic examination was performed after 24 h Gram staining from the culture to describe the shape of the cells and their mode of association.

Fermentative biochemical tests differentiate heterofermentative LAB from homofermentative LAB and focus on the production of CO_2_. To inoculate the studied strains, this test was performed in a tube containing MRS broth without meat extract and a Durham bell. After incubation at 30°C for 24 h, the presence of gas in the bell jar indicates heterofermentative metabolism [18].

The search for arginine dihydrolase was performed using M16BCP. LAB strains use lactose to acidify the medium, giving the colonies a yellow color. Other LAB strains can also use arginine to re-alkalinize the medium. This enzyme releases ammonia and citruline from the arginine and changes the color of the medium from yellow to purple.

LAB were inoculated and incubated at 30°C for 24 h on KMK (Kempler and Mc Kay) medium supplemented with potassium ferricyanide solution and a solution of ferric citrate; citrate fermentation resulted in the appearance of blue colonies; colonies unable to ferment citrate appeared white [19].

### Phenotypic and biochemical identification

Gram staining was performed using a standard bacteriological procedure.

### Extraction and identification of LAB strains by ARNr 16S sequencing

Bacterial genomic DNA was extracted using the GF-1 Nucleic Acid Extraction Kit (Vivantis Technologies Sdn Bhd, Selangor DE, Malaysia) according to the manufacturer’s instructions. The DNA extracted was stored at 4°C.

Polymerase chain reaction (PCR) amplification was achieved using the primer set of 16S rRNA gene (27F: 5′–AGA GTT TGA TCC TGG CTC AG–3′ and 1492R 5′–CCG TCA ATT CCT TTG AGT TT–3′) reaction mixture containing 1× PCR buffer (Solis Biodyne, Estonia), 1.5 mM magnesium chloride (Solis Biodyne), 0.2 mM of each dNTP (DeoxyNucleotide TriphosPhates) (Solis Biodyne).

The PCR reaction mixture consisted of 25 μL of master mix (1.25 U Taq DNA Polymerase and Solis Biodyne), 3 μL of DNA template, 5 μL of each primer, and 50 μL of distilled water. In this study, DNA concentrations were measured using a nanodrop spectrophotometer.

#### Agarose gel electrophoresis

After PCR, the PCR product was separated on a 1.5% agarose gel; a 100 bp DNA ladder was used as a DNA molecular weight marker. PCR products were electrophoresed, purified, and sent to a sequencing agency. We analyzed the generated sequences using BLAST (Basic Local Alignment Search Tool) software.

The purified PCR products were sequenced in the forward and reverse directions in separate reactions and in duplicate. Each reaction consisted of 40 µg template DNA, 2 µL of appropriate PCR primer, 10 µL of water, and 20 µL of BigDye Terminator v3.1 (BigDye™ Terminator v3.1 Cycle Sequencing Kit’s robust, highly flexible chemistry is ideal for *de novo* sequencing, resequencing, and finishing with PCR product, plasmid, fosmid, and BAC templates) includes 1 x 800μl tube of BigDye™ Terminator v3.1 Ready Reaction Mix, 1 tube M13 (-21) Primer, 1 tube pGEM Control DNA, 2 x 1mL tubes of 5X Sequencing Buffer). Ready Reaction Mix. To obtain a composite sequence, two forward and two reverse sequences for each sample were aligned using Bionumerics v3.5 (Applied Maths, Belgium). We visually assessed the quality of each sequence trace, edited, and removed poor-quality sequences. Organisms for each assay were identified by comparing consensus sequences with a database.

### Antimicrobial activity

Isolated LABs were tested for their antagonistic activity using two methods:

- The direct method brings the supernatant of the lactic strain, which produces the antimicrobial substance, into contact with the indicator strain. This test concerns strains previously selected for the production of antimicrobial substances. The strains were cultured in liquid MRS medium and incubated for 18 h. After incubation, the medium was centrifuged at 8000 t/min for 10 min, and the supernatant was retained. In a Petri dish containing the agar nutrient, the indicator strain was inoculated, wells were made by a part carrier, the wells received 100 μL of the supernatant of the strain to be tested, and the dishes were incubated for 24 h. Wells surrounded by a clear zone in the culture sheet of the tested strain and having a diameter >2 mm are considered positive.
- The indirect method brings the supernatant of the LAB strain into contact with the indicator strain. This test involved strains previously selected for the production of antimicrobial substances. These strains were cultured in liquid MRS medium and incubated for 18 h. Subsequently, the medium was centrifuged for 10 min at 5,009× *g* and the supernatant was retained. In a Petri dish containing nutrient agar, the indicator strain was inoculated into wells made by a part carrier. The wells were incubated for 24 h with 100 μL of the supernatant of the tested strain. Wells surrounded by a clear zone of the tested strain with a diameter >2 mm are considered positive.

*Escherichia coli*, *Staphylococcus aureus, Pseudomonas aeruginosa, Listeria ivanovii, Chryseobacterium joostei, Klebsiella* spp., and *Salmonella enterica serovar typhimurium* were used as indicator strains for antimicrobial activity.

### Inhibition due to the production of dihydrogen peroxide

This agent can be degraded by the enzyme catalase present in certain bacterial species, such as *S. aureus*, which leads to the production of dihydrogen peroxide H_2_O_2_, an inhibitor of bacterial growth [20].

### Inhibition due to organic acid production

Lactic acid plays a major role in inhibition by LAB. Strains were cultured in MRS-buffered liquid medium (0.1 M phosphate buffer pH 7).

### Physicochemical characterization of proteins

Certain physicochemical properties of bacteriocins give an idea of their classification. In particular, heat-resistant bacteriocins belong to Class III, whereas heat-resistant bacteriocins are class I or II [21]. Therefore, culture supernatants were treated to study the effect of different physicochemical parameters on bacteriocin activity. The well diffusion method was used for all tests conducted in this section.

### Aromatic power of the selected strains

Using sterile skim milk, the ability of strains to produce flavoring compounds can be demonstrated. Each tube containing sterile skim milk was inoculated with one of the following strains. After incubation at 30°C for 24–48 h, Vogues-Proskaeur (VP) I and VPII reagents were added and incubated for 24–48 h at 25°C [22]. The presence of aroma is indicated by the appearance of a red ring.

### Probiotic profiles (acidity tolerance, bile salt resistance, and bile salt hydrolysis) of the strains

Bacterial cells from the 18-h culture of the strains incubated in MRS agar broth were recovered by centrifugation and washed with sterile phosphate buffer solution (pH 7). Centrifugation and washing procedures were repeated 3 times, and the bacterial cells were recovered in sterile phosphate-buffered saline (PBS) adjusted to pH 2, 3, and 4. According to the counting method, the number of viable cells was determined after exposure to the acid state for 0 and 3 h at 37°C. After incubation at 30°C for 48 h [22], the resulting colony-forming unit (CFU)/mL values are expressed in log.

Bacterial cells from 18-h cultures were harvested by centrifugation, washed, and resuspended in (PBS; pH 8) supplemented with 0.5%, 1.0%, or 2.0% bile salts. The number of viable cells was determined after exposure to bile salts for 0 and 4 h at 37°C and incubation at 30°C for 48 h, according to the counting method. These values are expressed in log CFU/mL.

The bile salt hydrolysis test is based on the determination of the bile hydrolase enzyme that catalyzes the hydrolysis of bile salt. Petri dishes containing modified MRS prepared with 0.5% bile salt were inoculated with 0.1 mL of the strain culture and incubated at 30°C for 48 h.

### Pepsin resistance

Bacterial strain cells from the 18-h culture were harvested by centrifugation and washed with sterile PBS) at pH 8. Bacterial cells were finally resuspended in sterile PBS adjusted to pH 2 and 3 and supplemented with 3 mg/mL pepsin. According to the counting method, the number of viable cells was determined after exposure to pepsin for 0 and 3 h at 37°C, and after incubation at 30°C for 48 h. These values are expressed as log CFU/mL.

### Hydrophobicity

We evaluated the hydrophobicity according to the method described by Djeribi and Benredjem [23]. The 18-h bacterial pellet was recovered by cold centrifugation at 7,826× for 10 min, followed by two successive washes, and resuspended in PBS (pH 7). The initial optical density (OD) of the suspension (initial OD 600) was adjusted to approximately 10^8^ CFU/mL. 1 mL of xylene was added to 3 mL of the bacterial suspension. After incubation for 20 min at 25°C, this mixture was stirred using a vortex for 2 min and the OD of the aqueous phase (final OD 600) was measured. The difference in OD was obtained as a measure of the cell surface hydrophobicity (H%) calculated using the following equation:


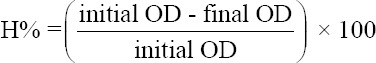


where OD is optical ensity.

## Results

### Phenotypic, genotypic, and characterization of isolated strains

The strains were isolated from 10 samples of fermented equine milk on MRS and M17 culture media, which showed 130 Gram+ and catalase isolates corresponding to LAB. Purification of these isolates reduced this number to 60 pure strains. We selected 31 of them that produced antimicrobial agents. Microscopic observations after Gram staining showed that these isolates were Gram+ and formed two groups: Rod-shaped and ovoid-shaped groups deposited in pairs or in short chains, respectively.

#### Fermentation profiles of selected LAB strains

The fermentation profiles of the six sugars were established using MRS BCP medium containing bromocresol purple (pH indicator). The yellow color indicates the fermentation of the carbohydrates, which causes acidification of the medium (Table-1).

**Table-1:** Physiological and biochemical characteristics of 21 LAB strains isolated from mare’s and donkey’s fermented milk.

Strains	Fermentative type	Temperature				NaCl		Hormone		pH			Fermentation profile					
				
		15°C	30°C	45°C	63.5°C/ 30 min	4%	6.5%	ADH	Citrate	4.8	6.5	9.6	Xylose	Sorbitol	Trehalose	Sucrose	Arabinose	Maltose
Mare’s milk																		
Lpseu	Homo	+	+	+	+	+	+	-	-	+	+	-	-	+	±	±	+	±
Lgra	Homo	+	+	-	+	+	+	-	+	+	+	-	-	+	-	+	-	-
Lfru	Homo	+	+	-	+	+	+	-	-	+	+	-	±	-	±	+	+	-
Wco	Hetero	+	+	-	-	+	-	+	+	-	+	-	+	-	±	±	+	+
Lcas	Homo	+	+	-	+	+	+	-	-	+	+	-	±	+	-	+	+	-
Lmes	Hetero	+	+	-	-	+	-	-	+	-	+	-	-	+	-	±	+	+
Lsp1	Hetero	+	+	-	-	+	-	-	+	-	+	-	-	+	-	±	+	+
Wcib	Hetero	+	+	+	-	+	-	-	+	-	+	-	±	+	+	+	+	+
Lpar	Hetero	+	+	-	+	+	+	-	-	+	+	-	±	+	-	+	+	+
Llac	Hetero	+	+	+	-	+	+	+	+	-	+	-	±	-	+	+	+	-
Donkey’s milk																		
Lpla	Hetero	+	+	-	-	+	+	+	+	-	+	-	-	+	+	+	-	-
Esp1	Hetero	+	+	-	-	+	+	+	+	-	+	-	±	-	-	-	-	-
Lpen	Hetero	+	+	-	-	+	+	+	+	-	+	-	-	-	±	+	±	-
Bsp2	Homo	+	+	-	+	+	+	+	+	+	+	-	+	+	-	+	±	±
Lmes	Homo	+	+	+	+	+	+	-	-	+	+	-	-	+	±	±	+	±
Bsp1	Homo	+	+	-	+	+	+	-	-	+	+	-	±	-	±	-	-	+
Lpar	Hetero	+	+	-	-	+	+	+	-	-	+	-	-	±	-	±	+	±
Llac	Hetero	+	+	+	-	+	+	+	+	-	+	-	±	-	+	+	+	-
Lcas	Homo	+	+	-	+	+	+	-	-	+	+	-	±	+	-	+	+	-
Bamy	Hetero	+	+	-	-	+	+	-	-	-	+	-	+	±	±	+	+	+
Bsaf	Homo	+	+	+	+	+	+	-	-	+	+	-	+	±	-	-	+	±

Symbols: +=Positive reaction, -=Negative reaction, ±=incomplete degradation, Lpseu=*Leuconostoc pseudomesenteroides*, Lgra=*Leuconostoc graviae*, Lfru=*Lactobacillus frumenti*, Wcon=*Weissella confusa*, Lcas=*Lactobacillus casei*, Lmes=*Lactobacillus mesenteroides*, Lsp1=*Lactobacillus* sp1, Wcib=*Weissella cibaria*, Lpar=*Lactobacillus paracasei*, Llac=*Leuconostoc lactis*, Lpla=*Lactiplantibacillus plantarum*, Esp1=*Enterococcus* sp1, Lpen=*Lactobacillus pentosus*, Bsp2=*Bacillus* sp2, Bsp1=*Bacillus* sp1, Bamy=*Bacillus amyloliquefaciens*, Bsaf=*Bacillus safensis*. 60% of the strains are homofermentative, which ferment sugar into lactate without producing

Gas and the others are heterofermentative, in which gas is released, which pushes the Durham bell upward.

On M16.BCP (Bromo cresol purple lactose agar is a non-selective medium), 51.6% of isolates were unable to hydrolyze arginine, indicating that LAB use lactose to acidify the medium. The colonies have a yellowish color, and the other isolates use arginine and re-alkalinize the medium, whitish color. The KMK medium was used to determine the ability to degrade citrate, which appears at low concentrations in milk, but it is still a key component in the development of fermented milk. Strains capable of fermenting citrate allow a reaction between ferric and potassium ferricyanide. Thus, our results show the formation of blue colonies in all tested isolates.

*Leuconostoc graviae*, *Leuconostoc lactis* (Llac), *Weissella cibaria* (Wcib), *Lactobacillus paracasei* (Lpar), and *Lactiplantibacillus plantarum* (Lpla) strains show clear resistance to fosfomycin (FF), oxacillin (Ox), and ampicillin, but their sensitivity to amikacin, clindamycin, and doxycycline is well-marked (Table-2). Antibiotic resistance is considered a prerequisite for selecting a probiotic strain [24–27]. The susceptibility and resistance of LAB to various antibiotics vary according to species and strains. Bacteria are highly adaptable and can develop antibiotic-resistance [25–28].

**Table-2:** Assessment of 14 lactic acid bacteria strains to 12 different types of antibiotics.

Antibiotics/Strains	Lpseu	Lgra	Llac	Lfru	Wcon	Lcas	Llac	Wcib	Lpla	Lpen	Lpar	Lcas	Bamy	Bsaf
Amoxicillin/Clavulanic Acid (Aug)	I	S	I	S	S	I	I	I	I	S	R	R	S	I
Clindamycin (Cd)	S	S	I	I	S	R	S	S	S	R	I	I	S	S
Tobramycin (Tob)	I	I	R	R	I	S	R	I	I	R	R	R	I	S
Amikacin (Ak)	I	I	R	R	I	S	S	I	S	R	S	R	I	S
Tetracycline (Te)	I	I	I	S	I	S	R	S	S	S	R	R	S	S
Doxycycline (Do)	S	S	S	S	S	R	S	S	R	I	S	R	I	R
Chloramphenicol (C)	S	S	S	I	R	I	S	S	I	S	R	S	S	R
Oxacillin (Ox)	R	R	R	R	R	I	I	R	R	R	R	I	S	I
Rifampicin (Ra)	I	S	I	R	I	S	S	I	S	I	R	S	I	S
Ampicillin (Amp)	R	R	R	I	R	S	R	R	R	I	R	I	I	R
Rapamycin (K)	I	I	R	S	S	I	S	S	R	R	S	R	R	S
Fosfomycin (FF)	R	R	R	S	R	S	R	R	R	I	R	S	R	S

Symbols: S=Sensitive, ≥21 mm, I=Intermediate, 16–20 mm, R=Resistant, <16 mm. Lpseu=*Leuconostoc pseudomesenteroide*s, Lgra=*Leuconostoc graviae*, Lfru=*Lactobacillus frumenti*, Wcon=*Weissella confusa*, Lcas=*Lactobacillus casei*, Wcib=*Weissella cibaria*, Lpar=*Lactobacillus paracasei*, Llac=*Leuconostoc lactis*, Lpla=*Lactiplantibacillus plantarum*, Bamy=*Bacillus amyloliquefaciens*, Lpen=*Lactobacillus pentosus*

LAB are naturally resistant to many antibiotics due to their structure and physiology [29, 30]. Moreover, according to Temmerman *et al*. [31], 68.4% of isolated probiotics have resistance to one or more antibiotics. *Lactobacillus* strains were resistant to kanamycin (81%), tetracycline (Te) (29.5%), erythromycin (12%), and chloramphenicol (C) (8.5%). About 38% of the *Enterococcus faecium* isolates were resistant to vancomycin. In most cases, this resistance is not transmissible; however, it is possible that the plasmid encoding antibiotic-resistance is transmitted to other strains. Therefore, strains with no resistance potential transfer were selected for this reason.

#### Taxonomic composition of bacterial communities in donkey milk samples

According to 16S rDNA sequencing, the taxonomic inference of donkey milk bacteria and the relative abundance of recovered sequences consisted of 10 genera and 50 isolates: *Acinetobacter schindleri* (2%), *Acinetobacter* spp. (5%, 4 isolates), *Aerococcus viridans* (3%), *Aerococcus* spp. (5 %, 3 isolates)*, Bacillus amyloliquefaciens* (5 %), *Bacillus safensis* (2%), *Bacillus* sp1 (2%), *Bacillus* sp2 (2%), *Bacillus* spp. (21%, 6 isolates), *Enterococcus* spp. (2%) *Enterococcus* spp. (6%, 6 isolates), Lpla (5%), Lpar (3%), *Lactobacillus casei* (Lcas) (2%), *Lactobacillus pentosus* (2%), *Leuconostoc mesenteroides* (4%), *Macrococcus* spp. (3%), *Staphylococcus epidermidis* (3%), *Staphylococcus haemolyticus* (2%), *Staphylococcus succinus* (2%), *Staphylococcus saprophyticus* (2%), *Staphylococcus* spp. (7%, 6 isolates), *Streptococcus* spp. (4%, 4 isolates), Wcib (2%), and unclassified (4%, 3 isolates). All these bacterial strains have previously been sequenced and studied from dairy products, such as donkey’s milk [6, 30, 31], goat’s milk [32], camel’s milk [33, 34], and sheep’s milk [35].

#### Taxonomic composition of the bacterial communities in mare milk samples

The bacterial microbiota composition at the genus and species levels of mare fermented milk consists of nine genera and 60 isolates: Lpar (6%), Lcas (2%), *Lactobacillus frumenti* (Lfru) (4%), *Lactobacillus* spp (26%, 14 isolates), *Lactococcus garvieae* (4%), *Leuconostoc gravieae* (5%), Llac (4%*)*, *L. mesenteroides* (4%), *Leuconostoc pseudomesenteroides* (Lpseu) (5%), *Leuconostoc* sp1 (2%), *Leuconostoc* spp (2%, 4 isolates), *Weissella confuse* (Wcon) (3%), *Rothia* spp (2%, 2 isolates), *Streptococcus parauberis* (2%), *Streptococcus* spp (3%, 5 isolates), *E. faecium* (4%), *Enterococcus* spp (6%, 7 isolates), *Acetobacter* spp (8%, 8 isolates), and unclassified (8%, 10 isolates).

The genera cited in this study are similar to those reported in previous studies by Hassaïne and Zadi-Karam Karam [33], Benmechernene *et al*. [34]. In raw mare’s milk, 286 genera were identified; however, *Lactobacillus* and *Staphylococcus* genera (33.1 and 32.9%, respectively) comprised the microbiota. These results were slightly discrepant with those reported in [36] and could reflect differences in the geographical origins of the samples, sampling seasons, and other environmental factors.

#### Inhibition due to the production of organic acids or hydrogen peroxide

After elimination of the effect of organic acids from the supernatant of the strains, 40% of the strains lost their inhibitory activity (Table-3). The observation that 60% of the strains inhibited the indicator strains confirms the existence of other antibacterial substances, such as hydrogen peroxide and bacteriocins (Table-4).

**Table-3:** Antimicrobial activity of lactic strains by direct method.

Pathogen bacteria/Strains	Lpseu	Lgra	Llac	Lfru	Wcon	Lcas	Llac	Lsp1	Wcib	Lpar	Lpen	Bsp2	Lmes	Esp1	Lpa	Bamy	Lsaf
*Escherichia coli*	+	+	-	+	+	+	+	-	+	+	+	+	-	+	+	+	+
*Staphylococcus aureus*	+	+	+	+	+	-	+	+	+	+	+	+	+	+	+	-	+
*Listeria ivanovii*	+	+	+	+	+	+	+	+	+	+	-	+	+	-	+	-	+

Symbols: -=No reaction, +=The number is the diameter of the well. Lpseu=*Leuconostoc pseudomesenteroide*s, Lgra=*Leuconostoc graviae*, Lfru=*Lactobacillus frumenti*, Wcon=*Weissella confusa*, Lcas=*Lactobacillus casei*, Wcib=*Weissella cibaria*, Lpar=*Lactobacillus paracasei*, Llac=*Leuconostoc lactis*, Lpla=*Lactiplantibacillus plantarum*, Bamy=*Bacillus amyloliquefaciens*, Lpen=*Lactobacillus pentosus*, Lmes=*Lactobacillus mesenteroides*, Esp1=*Enterococcus* sp1, Bsp2=*Bacillus* sp2

**Table-4:** Antimicrobial activity of lactic strains by indirect method.

Pathogen bacteria/Strains	Lpseu	Lgra	Llac	Lfru	Wcon	Lcas	Llac	Lsp1	Wcib	Ppla	Lpen	Bsp2	Lmes	Bsp1	Esp1	Lpar	Lcas	Bamy	Lsaf
*Escherichia coli*	15	11	10	13	11	12	11	9	11	13	14	11	13	-	15	14	6	10	12
*Staphylococcus aureus*	-	15	9	10	13	21	16	10	12	8	12	10	10	16	13	12	12	9	12
*Pseudomonas aeruginosa*	12	15	-	12	12	18	18	17	17	13	-	13	14	17		16	12	12	12
*Listeria ivanovii*	12	14	18	15	16	11	15	15	11	12	21	15	13	12	9	20	12	8	19
*Salmonella enterica serovar typhimurium*			-		-	13	10		-	-	-	-	-		-	16		-	
*Klebsiella spp*	-	10	-		-	10	13	13	15	-	12	12	13	12	13	-		-	10
*Chryseobacterium joostei*	15	11	10	13	14	11	11	9	11	13	12	11	13	14	10	12	13	6	15

Symbols: -=No reaction, The number is the diameter of the well. Lpseu=*Leuconostoc pseudomesenteroide*s, Lgra=*Leuconostoc graviae*, Lfru=*Lactobacillus frumenti*, Wcon=*Weissella confusa*, Lcas=*Lactobacillus casei*, Wcib=*Weissella cibaria*, Lpar=*Lactobacillus paracasei*, Llac=*Leuconostoc lactis*, Lpla=*Lactiplantibacillus plantarum*, Bamy=*Bacillus amyloliquefaciens*, Lpen=*Lactobacillus pentosus*, Lmes=*Lactobacillus mesenteroides*, Esp1=*Enterococcus* sp1, Bsp2=*Bacillus* sp2, Bsp1=*Bacillus* sp1

The involvement of hydrogen peroxide in the inhibition of LAB has been reported [37], which confirmed that when H_2_O_2_ is released in sufficient concentrations, it can inhibit certain contaminants. The antimicrobial activity of lactic strains buffered at pH 7 is shown in Table-5, and the biotechnological characteristics of 19 selected strains are presented in Table-6.

**Table-5:** Antimicrobial activity of lactic strains buffered at pH 7.

Pathogen bacteria/Strains	Lpseu	Lgra	Llac	Lfru	Wcon	Lcas	Llac	Lsp1	Wcib	Ppl	Lpen	Bsp2	Lmes	Bsp1	Esp1	Lpar	Lcas	Bamy	Bsaf
*Escherichia coli*	11	5	10	9	8	14	7	10	13	12	10	9	11	11	11	11	10	5	5
*Staphylococcus aureus*	13	16	11	13	13	11	6	14	10	18	10	8	8	10	10	13	9	16	16
*Pseudomonas aeruginosa*	9	10	3	11	10	6	12	15	16	17	14	14	12	6	13	9	3	10	10
*Listeria ivanovii*	9	10	5	7	8	8	8	14	11	13	6	12	9	8	12	9	/	11	10
*Salmonella enterica serovar typhimurium*	/	6	/	/	6	/	/	9	/	6	/	11	/	/	10	6	/	/	6
*Klebsiella spp.*	/	/	/	9	6	/	6	6	/	6	6	/	11	/	/	6	/	/	/
*Chryseobacterium joostei*	13	-	-	13	13	11	6	14	10	18	10	8	8	10	10	13	/	16	16

Lpseu=*Leuconostoc pseudomesenteroide*s, Lgra=*Leuconostoc graviae*, Lfru=*Lactobacillus frumenti*, Wcon=*Weissella confusa*, Lcas=*Lactobacillus casei*, Wcib=*Weissella cibaria*, Lpar=*Lactobacillus paracasei*, Llac=*Leuconostoc lactis*, Lpla=*Lactiplantibacillus plantarum*, Bamy=*Bacillus amyloliquefaciens*, Lpen=*Lactobacillus pentosus*, Lmes=*Lactobacillus mesenteroides*, Esp1=*Enterococcus* sp1, Bsp2=*Bacillus* sp2, Bsp1=*Bacillus* sp1, Bsaf=*Bacillus* safensis

**Table-6:** Biotechnological characteristics of 19 selected strains.

Strains	Lpseu	Lgra	Llac	Lfru	Wcon	Lcas	Llac	Lsp1	Wcib	Ppla	Lpen	Bsp2	Lmes	Bsp1	Esp1	Lpa	Lcas	Bamy	Bsaf
Aroma production	-	+	+	+	+	+	-	+	+	+	+	+	+	-	+	+	+	+	-
EPS production	+	-	+	+	-	-	-	+	+	-	-	+	-	+	+	-	-	+	-
Proteolytic activity	15	12	13	11	-	6	20	17	11	13	-	14	13	-	28	-	-	17	11
Lipolytic activity	-	-	-	-	-	-	-	-	-	-	-	-	-	-	-	-	-	-	-

Lpseu=*Leuconostoc pseudomesenteroide*s, Lgra=*Leuconostoc graviae*, Lfru=*Lactobacillus frumenti*, Wcon=*Weissella confusa*, Lcas=*Lactobacillus casei*, Wcib=*Weissella cibaria*, Lpar=*Lactobacillus paracasei*, Llac=*Leuconostoc lactis*, Lpla=*Lactiplantibacillus plantarum*, Bamy=*Bacillus amyloliquefaciens*, Lpen=*Lactobacillus pentosus*, Lmes=*Lactobacillus mesenteroides*, Esp1=*Enterococcus* sp1, Bsp2=*Bacillus* sp2, Bsp1=*Bacillus* sp1, Bsaf=*Bacillus* safensis

#### Flavoring power

Some LAB produce many aromatic compounds from different substrates that contribute to the organoleptic properties of fermented milk or cheese [38]. The majority of aromatic compounds originate from citrate metabolism; acetoin and diacetyl are the most important compounds. The metabolism of citrate and lactose leads to the production of diacetyl, acetoin, and CO_2_, which contributes to the aromatic and textural properties of products [38, 39].

**Figure-1:**
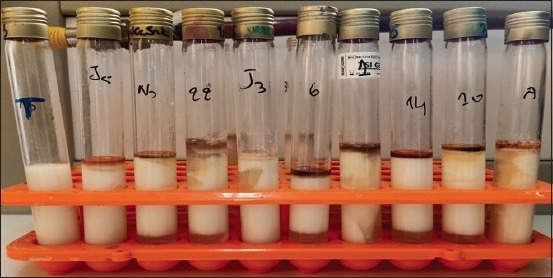
Production of aromas (acetoin) by the 9 experimented strains. T=Witness.

Lpla, Lcas, Lpar, Wcon, Wcib, *L. mesenteroides*, Llac, and *Lactobacillus* sp1 have the capacity to produce aromas (acetoin), as confirmed by the presence of a red ring on the surface of the tube (Figure-1). Lfru (J3) cannot produce aroma.

#### Probiotic skills of the selected strains

Tolerance to acidity of the strains

The acid tolerance of bacteria is important not only for gastro-resistant stasis but also for their use as dietary supplements, allowing strains to survive longer in acid-rich environments without compromising human health. Although the stomach pH can increase to 6.0 or higher after food intake, it is generally between 2.5 and 3.5 [40]. Prolonged exposure of the strains to acidic conditions similar to those of the human stomach was carried out by exposure to different pH values (2, 3, and 4) for 3 h. Table-7 illustrates the results obtained. The pH-resistance results showed that *L. mesenteroides*, Lcas, Llac, and Wcib were not viable at pH 2 but survived at pH 3 and pH 4 after 3 h of exposure, whereas Lpar, Wcib, and Lpla were viable at pH 2, 3, and 4, respectively. These results are partly in agreement with previous data that reported the absence of bacterial viability at pH 2.0 [40]. *L. mesenteroides* strain was not viable at pH 2 but was viable at pH 3 and 4 [34].

**Table-7:** Effect of different acidic pH on strains viability (Standard variations).

Acidity at 0 h													

Stra_ins	Llac	Lgra	Bsp1	Ppla	Lpar	Bsaf	Lmes	Wcib	Esp1	Wcon	Lsp1	Llac	Lfru
pH 2	0	0.005773	0	0	0.005773	0.005773	0	0.005773	0	0.005773	0.005773	0.005773	0
pH 3	0.005773	0.005773	0.005773	0	0.005773	0.005773	0.005773	0.005773	0.005773	0.005773	0.005773	0.005773	0.005773
pH 4	0.005773	0.005773	0.005773	0.005773	0.005773	0.005773	0.005773	0.005773	0.01	0.01	0.01	0.01	0.005773

**Acidity at 3h**													

**Strains**	**Llac**	**Lgra**	**Bsp1**	**Ppla**	**Lpar**	**Bsaf**	**Lmes**	**Wcib**	**Esp1**	**Wcon**	**Lsp1**	**Llac**	**Lfru**

pH 2	0	0.005773	0	0	0.005773	0.005773	0	0.005773	0	0.005773	0.005773	0.005773	0
pH 3	0.005773	0.005773	0.005773	0	0.005773	0.005773	0.005773	0.005773	0.005773	0.005773	0.005773	0.005773	0.005773
pH 4	0.005773	0.005773	0.005773	0.005773	0.005773	0.005773	0.005773	0.005773	0.01	0.01	0.01	0.01	0.005773

Llac=*Leuconostoc lactis*, Lgra=*Leuconostoc graviae*, Bsp1=*Bacillus* sp1, Lpar=*Lactobacillus paracasei*, Bsaf=*Bacillus* safensis, Lmes=*Lactobacillus mesenteroides*, Wcib=*Weissella cibaria*, Esp1=*Enterococcus* sp1, Lsp1=Lactobacillus sp1, Wcon=*Weissella confusa*

Resistance to gastric acidity

Bacterial survival in gastric juice clearly depends on the ability of bacteria to tolerate low pH. Breakthrough time may vary from 1 to 4 h depending on the individual and the diet. Therefore, probiotic strains should withstand a pH of 2.5 in a culture medium for 4 h [40].

Resistance of bile salt

Bile salt tolerance in the small intestine is an important factor contributing to probiotic survival. Bacteria that survive acidic conditions in the stomach must then synthesize bile salts released in the duodenum after ingestion of fatty foods. Bacteria can reduce the emulsifying effect of bile salts by hydrolyzing them with hydrolases, thereby decreasing their solubility [41–44].

Antibiotic resistance

LAB are naturally resistant to a number of antibiotics because of their structure and physiology. According to Temmerman *et al*. [31], 68.4% of isolated probiotics have resistance to one or more antibiotics. *Lactobacillus* strains were resistant to kanamycin (81%), Te (29.5%), erythromycin (12%), and C (8.5%). 38% of *E. faecium* isolates were resistant to vancomycin. In most cases, resistance is not transmissible; however, plasmid encoding antibiotic-resistance may be transmitted to other species and genera. This is a significant reason why strains lacking resistance transfer potential are selected.

The European authorities have recently concluded that certain bacteria used for food production may pose a risk to human and animal health if they contain strains with inheritable resistance genes. Therefore, before initiating a probiotic culture, it is important to verify that the bacterial strains used do not carry transmissible antibiotic-resistance genes [43].

Strain resistance and bile salt hydrolase activity

Bacterial resistance to bile salts is an essential criterion for the selection of probiotic strain. The small intestine and colon are the first colonization niches of the host organism [30]. Table-8 presents the results of the bile salt resistance tests.

**Table-8:** Effect of different concentrations of bile salts on strains (Standard deviation).

Bile salt at 0 h													

Strains	Llac	Lgra	Bsp1	Ppla	Lpar	Bsaf	Lmes	Wcib	Esp1	Wcon	Lsp1	Llac	Lfru
0.5%	0	0.005773	0.005773	0.005773	0.005773	0.005773	0.005773	0.005773	0.005773	0	0.005773	0.005773	0.005773
1%	0	0.005773	0.005773	0.005773	0.011547	0.005773	0	0.005773	0.005773	0	0.005773	0.005773	0.005773
2%	0.005773	0.005773	0.005773	0.005773	0.011547	0.005773	0.005773	0.005773	0.005773	0.005773	0.005773	0.005773	0.005773

**Bile salt at 4 h**													

**Strains**	**Llac**	**Lgra**	**Bsp1**	**Ppla**	**Lpar**	**Bsaf**	**Lmes**	**Wcib**	**Esp1**	**Wcon**	**Lsp1**	**Llac**	**Lfru**

0.5%	0.011547	0.015275	0.017320	0.017320	0.005773	0.005773	0	0.005773	0.005773	0.005773	0.005773	0.005773	0.005773
1%	0.01	0.017320	0.015275	0	0.005773	0.005773	0.005773	0.005773	0.005773	0.01	0.005773	0.005773	0.005773
2%	0.03	0.011547	0.025166	0.005773	0.011547	0.005773	0.005773	0.005773	0.011547	0.005773	0.005773	0.005773	0.005773

Llac=*Leuconostoc lactis*, Lgra=*Leuconostoc graviae*, Bsp1=*Bacillus* sp1, Lpar=*Lactobacillus paracasei*, Bsaf=*Bacillus* safensis, Lmes=*Lactobacillus mesenteroides*, Wcib=*Weissella cibaria*, Esp1=*Enterococcus* sp1, Lsp1=Lactobacillus sp1, Wcon=Weissella confuse

Exposure of strain cultures to different concentrations of bile salts moderately affected strain viability, and no hydrolyzed bile salts were detected in the strain. These results are similar to those obtained from the previous studies by Hosseini *et al*. [22], and Benmechernene *et al*. [34].

Pepsin resistance

Protein digestion begins in the stomach, where pepsin is released by the main gastric cells in the form of a precursor, pepsinogen II, which is activated into pepsin under the action of hydrochloric acid and has an optimal pH between 1 and 2. We evaluated the survival of the strains in the presence of 3 mg/mL of pepsin at pH 2 and 3 (Table-9). Exposure of pepsin at pH 2 had no observable effect, but it was not viable after 3 h of incubation. Exposure to pepsin at pH 3 revealed the viability of all tested strains and confirmed their resistance to pepsin. It has been confirmed that these strains are also resistant to the effects of pepsin [34].

**Table-9:** Effect of pepsin on strains viability at pH 2 and pH 3 (Standard deviation).

Pepsin at 0 h													

Strains	Llac	Lgra	Bsp1	Ppla	Lpar	Bsaf	Lmes	Wcib	Esp1	Wcon	Lsp1	Llac	Lfru
pH 2	0.005773	0.011547	0.005773	0.005773	0.005773	0.005773	0.01	0	0.005773	0.005773	0	0.005773	0.005773
pH 3	0.005773	0.005773	0	0.005773	0	0.005773	0	0.005773	0	0.005773	0.005773	0.005773	0.005773

**Pepsin after 3 h**													

**Strains**	**Llac**	**Lgra**	**Bsp1**	**Ppla**	**Lpar**	**Bsaf**	**Lmes**	**Wcib**	**Esp1**	**Wcon**	**Lsp1**	**Llac**	**Lfru**

pH 2	0.0057	0	0.005773	0	0.005773	0.005773	0.005773	0.005773	0	0	0	0.005773	0
pH 3	0	0.005773	0.005773	0.005773	0.011547	0	0	0.005773	0	0	0.005773	0.005773	0.005773

Llac=*Leuconostoc lactis*, Lgra=*Leuconostoc graviae*, Bsp1=*Bacillus* sp1, Lpar=*Lactobacillus paracasei*, Bsaf=*Bacillus* safensis, Lmes=*Lactobacillus mesenteroides*, Wcib=*Weissella cibaria*, Esp1=*Enterococcus* sp1, Lsp1=Lactobacillus sp1, Wcon=*Weissella confusa*

Hydrophobicity of the selected strains

**Figure-2:**
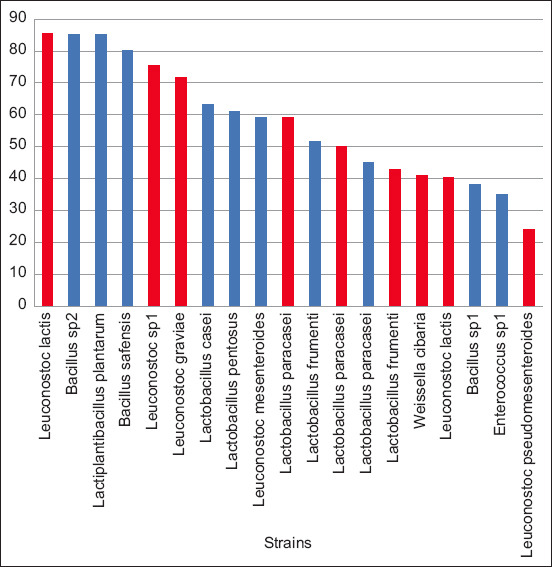
Percentage of the strain’s hydrophobicity (red=mare milk, blue=donkey milk).

Assessment of cell surface hydrophobicity of LAB strains against xylene reflects the colonization potential of ferments with intestinal mucus [45]. The distribution of cells between the aqueous and xylene phases resulted from the hydrophobic interaction between bacteria and hydrocarbons. The percentages of *L. mesenteroides* (85.49%) and Lpla (85.07%) were significantly higher for the 19 strains (Figure-2). The high percentage indicates the good selectivity of the membrane surfaces. Lpseu (24.10%) showed the lowest value. These results are significantly higher than those for other probiotic strains such as *Leuconostoc mesenterodes* (42.9%) [44], *Lactococcus acidophilus* (38.1%), *Lactococcus casei* (24.1%), and *Lactococcus lactis* (31.3%) [44].

The tested strains have good hydrophobicity and selectivity to membrane surfaces. Following Zago *et al*. [27], *L. lactis* ssp. diacetylactis C23 had the highest value (63.58%), whereas the lowest value (11.37%) was recorded with the F1 ferment. A significant difference was observed between the pure strains and mixed ferments (p < 0.05). Our results corroborate those of Ly-Chatain *et al*. [46], who found a hydrophobicity of 40% for *L. lactis* ssp. *lactis* biovar. diacetylactis and *Streptococcus salivarius* ssp. *thermophilus* strains. The hydrophobicity of the cell wall facilitates the first contact between microorganisms and host cells. It appears to be a factor that helps adhesion, but it does not contribute to good adhesion [45, 46]. According to Guglielmotti *et al*. [47], *Lactobacillus* species exhibit hydrophobicity varying between 5% and 63%. In another study by Pan *et al*. [48], the hydrophobicity of 23 strains of *Bifidobacterium* was between 32% and 37%.

It is important to consider the pathogenic potential in all technological and functional aspects versus the whole genome analysis-based pathogenic potential for their application in food and health. Following Abriouel *et al*. [49], the application of Wcon and Wcib strains as starter cultures or as probiotics should be approached with caution by carefully selecting strains that lack pathogenic potential. The same applies to strains of *Enterococcus* species. In this sense, *Weissella* and *Enterococcus* strains performed poorly in the tests (Figure-2) and are therefore not recommended as probiotics in this study.

The tested strains have good hydrophobicity related to good selectivity for membrane surfaces. Following Zago [27], the highest value (63.58%) recorded was with *Lactococcus lactis ssp*. *diacetylactis* C23 and the lowest was recorded with the F1 ferment (11.37%). The difference recorded between the pure strains and the mixed ferments was significant (P < 0.05). Our results corroborate those of Ly-Chatain [50], who found a hydrophobicity of 40% for strains of *Lc*. *lactis* ssp. *lactis biovar. diacetylactis* and *Streptococcus salivarius* ssp. *thermophilus*. The hydrophobicity of the cell wall is a physicochemical property that facilitates the first contact between microorganisms and host cells. It seems to be a factor that helps adhesion, but it does not contribute to good adhesion [50-51]. According to Guglielmotti *et al*. [52], *Lactobacillus* species exhibit hydrophobicity that varies between 5% and 63%. Furthermore, in another study by Pan *et al*. [53], the hydrophobicity of 23 strains of *Bifidobacterium* was between 32% and 37%. It is important to take in consideration the pathogenic potential in all technological and functional aspects versus whole genome analysis-based pathogenic potential for their application in food and health. Following Abriouel *et al*. [54], the application of *Weissella* *confusa* and *W. cibaria* strains as starter cultures or as probiotics should be approached with caution, by carefully selecting strains that lack pathogenic potential. The same holds for strains of genus Enterococcus. In this sense, the probiotics skills of *Weissella* and *Enterococcus* strains have a low performance in the tests (Figure-2) and are not recommended as probiotics in this work.

## Conclusion

Thirty-one strains of LAB were isolated from the fermented milk of donkeys and mares and identified by phenotypic characterization and 16S rDNA sequencing. Antibacterial activity tests showed a significant inhibition profile against pathogenic bacteria for all strains. Inhibitory agent of 19 important LAB strains was determined to be bacteriocin substance of a proteinaceous nature.

Technological tests of 19 best-performing strains producing bacteriocin have shown that Lpla and *L. mesenteroides* are the most suitable strains for industrial use because of their effective proteolytic activity and good acidifying power.

The 19 selected strains were non-hemolytic and their resistance to antibiotics (FF, Ox, and kanamycin) was well demonstrated in the laboratory.

This study of the probiotic profile suggests that the tested strains could be used as probiotics because of their aggregation properties, hydrophobic character, and tolerance to various biological barriers such as acids (pH 3 and 4), bile salts (0.5%, 1%, and 2%), and pepsin (3 mg/mL at pH 3), which confirmed their ability to survive in extreme conditions of the digestive tract.

An important number of species identified in this study have previously been detected in donkey and mare milk; however, their importance for probiotic activity and biotechnological potential in Algeria is highlighted. These results provide insight into the diversity of microorganisms present in the highly selective ecosystem of fermented donkey and mare milk.

## Data Availability

The datasets used in this study are available from the corresponding author on reasonable request.

## Authors’ Contributions

FB: Conceptualization, methodology, conducted the experiment and analyzed the data, and wrote manuscript–review. KB: Conceptualization, data validation, review, and editing manuscript. OK: Visualization supervision, data validation, review, and editing of manuscript. All authors have read, reviewed, and approved the final version of the manuscript.
